# Gene expression changes associated with trajectories of psychopathology in a longitudinal cohort of children and adolescents

**DOI:** 10.1038/s41398-020-0772-3

**Published:** 2020-03-17

**Authors:** Vanessa Kiyomi Ota, Marcos Leite Santoro, Leticia Maria Spindola, Pedro Mario Pan, Andressa Simabucuro, Gabriela Xavier, Tamiris Vieira-Fonseca, Evelin Aline Zanardo, Felipe Rodolfo Camargo dos Santos, Julia Luiza Schäfer, Leslie Domenici Kulikowski, Pedro A. F. Galante, Paula Fontes Asprino, Elisa Brietzke, Rodrigo Grassi-Oliveira, Luis Augusto Rohde, Euripedes Constantino Miguel, Ary Gadelha, Jair Jesus Mari, Rodrigo Affonseca Bressan, Giovanni Abrahao Salum, Sintia Iole Belangero

**Affiliations:** 1 LiNC - Interdisciplinary Laboratory of Clinical Neurosciences, Universidade Federal de São Paulo (UNIFESP), São Paulo, Brazil; 2 Post-Graduation Program in Psychiatry and Medical Psychology, UNIFESP, São Paulo, Brazil; 3 National Institute of Developmental Psychiatry for Children and Adolescents (INPD), São Paulo, Brazil; 4 Genetics Division, Department of Morphology and Genetics, UNIFESP, São Paulo, Brazil; 5 Laboratorio de Citogenomica, Departamento de Patologia, Faculdade de Medicina da Universidade de Sao Paulo (FMUSP), Sao Paulo, Brazil; 6 Centro de Oncologia Molecular, Hospital Sírio-Libanês, São Paulo, Brazil; 7 Universidade de Sao Paulo (USP), Sao Paulo, Brazil; 8 Department of Psychiatry, Hospital de Clínicas de Porto Alegre, Universidade Federal do Rio Grande do Sul (UFRGS), Porto Alegre, Brazil; 9 Kingston General Hospital and Providence Care Hospital, Department of Psychiatry, School of Medicine, Queen’s University, Kingston, ON Canada; 10 Centre for Neuroscience Studies (CNS), Queen’s University, Kingston, ON Canada; 11 Medicine and Medical Sciences Graduate Program, School of Medicine, Brain Institute (InsCer), Pontifical Catholic University of Rio Grande do Sul, Porto Alegre, Brazil; 12 Laboratórios de TDAH e Psiquiatria do Desenvolvimento, Hospital de Clínicas de Porto Alegre, Universidade Federal do Rio Grande do Sul (UFRGS), Porto Alegre, Brazil; 13 Department of Psychiatry, FMUSP, São Paulo, Brazil

**Keywords:** Biomarkers, Psychiatric disorders

## Abstract

We aimed to identify blood gene expression patterns associated to psychopathological trajectories retrieved from a large community, focusing on the emergence and remission of general psychiatric symptoms. Hundred and three individuals from the Brazilian High-Risk Cohort Study (BHRCS) for mental disorders were classified in four groups according to Child Behavior Checklist (CBCL) total score at the baseline (w0) and after 3 years (w1): low–high (L–H) (*N* = 27), high–low (H–L) (*N* = 12), high–high (H–H) (*N* = 34) and low–low (L–L) groups (*N* = 30). Blood gene expression profile was measured using Illumina HT-12 Beadchips, and paired analyses comparing w0 and w1 were performed for each group. Results: 98 transcripts were differentially expressed comparing w0 and w1 in the L-H, 33 in the H–L, 177 in the H–H and 273 in the L–L. Of these, 66 transcripts were differentially expressed exclusively in the L–H; and 6 only in the H–L. Cross-Lagged Panel Models analyses revealed that RPRD2 gene expression at w1 might be influenced by the CBCL score at w0. Moreover, *COX5B, SEC62*, and *NDUFA2* were validated with another technique and were also differentially regulated in postmortem brain of subjects with mental disorders, indicating that they might be important not only to specific disorders, but also to general psychopathology and symptoms trajectories. Whereas genes related to metabolic pathways seem to be associated with the emergence of psychiatric symptoms, mitochondrial inner membrane genes might be important over the course of normal development. These results suggest that changes in gene expression can be detected in blood in different psychopathological trajectories.

## Introduction

Mental disorders lack of validity and poor diagnosis stability is a major concern, especially in samples from young patients. Children and adolescents with mental disorders have a higher risk to present psychiatric problems during adulthood. Moreover, there are several transitions of symptomatic presentations over development^1^, and investigating these trajectories would help to develop prevention and early intervention policies for mental disorders.

Comorbidity levels are high among mental disorders^2^ and this may reflect the shared underlying etiologic factors, such as genetic factors. The heritability estimates of mental disorders vary from 40 to 80%^3,4^, and family studies suggest strong genetic correlations between several groups of these illness^5,6^. Recent studies have also shown that mental disorders share molecular mechanisms^7^, leading to a high genetic correlation among them. The most recent study from the Cross-Disorder Group of the Psychiatric Genomics Consortium identified 109 pleiotropic loci affecting multiple of them and these genes play major roles in neurodevelopmental processes, among others^8^. Another study showed that polygenic risk scores calculated for major depressive disorder (MDD), schizophrenia and attention deficit/hyperactivity disorder (ADHD) influence developmental trajectories of depression in youth, indicating that genetic liabilities to different mental disorders may affect developmental trajectories^9^.

Notably, overlapping patterns of gene expression among mental disorders were also observed. Investigating whole blood gene expression across adult and childhood ADHD, autism spectrum disorders (ASD), MDD, and healthy controls, De Jong et al.^10^ identified two immune-related gene co-expression modules inversely correlated with MDD and adult ADHD disease status and one module positively correlated with both MDD and childhood ADHD status^10^. In another study, Gandal et al.^11^ analyzed published gene-expression microarray studies of the cerebral cortex across five major neuropsychiatric disorders: ASD, schizophrenia, bipolar disorder (BD), MDD and alcoholism. Modules enriched for glial cell differentiation, fatty-acid metabolism, neuronal or mitochondrial pathways were shared across ASD, schizophrenia, and bipolar disorder^11^. These overlapping patterns of gene expression indicate the importance of investigating general psychiatric symptoms instead of specific mental disorders. We hypothesize that some genes have their expression affected by the course of general psychiatric symptoms. However, a comprehensive effort to investigate the association of gene expression and general psychiatric symptoms over the course of development has not been performed. To contribute to this issue, here we identify blood gene expression patterns associated to trajectories of psychopathology retrieved from a large community cohort, focusing on the emergence and remission of general psychiatric symptoms. We leverage our study by carefully analyzing selected subjects from the Brazilian High-Risk Cohort Study (BHRCS), a large community study with rich genotypic and phenotypic data. Considering that genetic factors and symptoms on mental disorders transcend diagnostic boundaries, investigating general psychiatric symptoms may help to obtain a more comprehensive picture. Moreover, analyzing these symptoms and gene expression patterns in a longitudinal design instead of a cross-sectional study might reveal clues about why overall symptoms emerge and disappear over the course of development.

## Materials and methods

### Study procedures and participant selection

We selected a subsample from a large prospective community school-based study in Brazil (*n* = 2512), the Brazilian High-Risk Cohort Study (BHRCS) for mental disorders. Briefly, the cohort included families recruited in two large Brazilian cities, Sao Paulo and Porto Alegre, and was assessed in two time points: wave 0—w0 and after three years of follow-up—w1. We collected biological data for a subsample of 621 individuals. Of these, 319 blood samples were available for both w0 and w1. After classifying the subjects into groups according to psychopathology, as described below, and selecting only samples from Sao Paulo, our final study sample was composed of 103 subjects. The Research Ethics Committee approved the research protocol. Parents provided written informed consent before the inclusion and children also provided written informed consent. The cohort characteristics and study design are detailed elsewhere^12^.

Dimensional psychopathology was assessed using the Child Behavior Checklist (CBCL)^13^, which was administered to the children’s biological parents. The CBCL is a broadly used inventory that provides parent-report information on a wide array of behavioral problems in youth, composed by 120 items rated as not true (0), somewhat or sometimes true (1), or very true or often true (2). CBCL total score was used to measure general psychiatric symptoms in children, considering that it comprises Internalizing behavior problems, which include withdrawn, somatic complains and anxious/depressed, and externalizing behavior problems, that correspond to delinquent behavior and aggressive behavior. Blood samples were also collected in the same day.

For the present study, we classified the participants into four groups according to total CBCL scores: low–high (L–H): those that had an increase in psychopathology at the end of the three years (CBCL at *w*0 ≤ 30 and CBCL at *w*1 > 30); high–low (H–L): those with a decrease in psychopathology at *w*1 moment (CBCL at *w*0 > 30 and CBCL at *w*1 ≤ 30); low–low (L–L) and high–high (H–H): individuals that maintained low (CBCL at *w*0 and *w*1 ≤ 30) or high (CBCL at *w*0 and *w*1 > 30) general psychopathology, respectively, in both waves. The CBCL threshold (30) was chosen based on a receiver operating characteristic (ROC) curve analysis (*n* = 2512) using CBCL score as a predictor of any mental disorder using the Development and Well Being Behavior Assessment (DAWBA), rated by trained psychiatrists. A CBCL threshold of 30 was able to predict any mental disorder based on DAWBA with a sensitivity of 75.6%, a specificity of 73.7% (Younden’s *J* = 0.019).

In addition, to fulfill the criteria, the L–H group should present an increase of at least 15 points (correspondent to 0.5 SD) in total CBCL score (ΔCBCL = CBCLw1—CBCLw0; ΔCBCL ≥15). Similarly, the H-L group should include those a decrease of at least 15 points in CBCL score (ΔCBCL ≤ −15). On the other hand, L–L group and H–H group, should have a variation of −15 < ΔCBCL < 15. These criteria were also used in a previous study^14^.

Using these criteria, we selected those with RNA samples available at both waves (*w*0 and *w*1) and from Sao Paulo, to avoid site effects: 27 L–H, 12 H–L, 34 H–H subjects and 30 L–L, totaling 103 subjects.

### Genetic analyses

#### RNA preparation

Blood was collected into PAXgene RNA tubes (BD) and RNA was isolated using PAXgene blood RNA kit (Qiagen). Quality and quantity were assessed using NanoDrop^TM^ 1000 Spectrophotometer (ThermoFisher Scientific) and Qubit 2.0 fluorometer with Qubit RNA BR Assay Kit (ThermoFisher Scientific), respectively. Samples presented 260/280 > 2.0 and mean concentration of 161.67 ng/µL (min = 21, max = 422, SD = 70.58). DNA contamination was verified in 1.5% agarose gel and RNA integrity was assessed using Agilent 2100 Bioanalyzer and RNA 6000 Nano Kit (Agilent). Samples presented mean RNA Integrity Number (RIN) of 8.65 (min = 6.7, max = 9.8, SD = 0.62).

#### Gene expression analyses

A total of 200 ng of RNA was used with Illumina® Total Prep™ RNA Amplification Kit (Life Technologies) to synthesize cRNA, which was hybrized to Human HT-12 v4 Expression BeadChips. Paired samples (*w*0 and *w*1) were included in the same chip, but groups were randomly allocated into each chip. The investigator was blinded to the group allocation during the experiment. BeadChips were scanned using the Illumina iScan System (San Diego, California, USA), and all the experiments were performed according to the manufacturer’s protocol.

##### Initial quality control and processing probes and samples

The results were downloaded from the iScan and preanalyzed using the GenomeStudio software. We checked the average signal and the number of detected genes for each sample.

Data was imported to R (https://www.r-project.org/) and quality control was performed using lumi package^15^, ending up with 6,322 probes with high-quality data available for further analyses. Then, we performed a background correction using the Maximum Likelihood Estimation (MLE) of the Model-Based Background Correction R package^16^. To ensure that the different BeadChips are comparable among each other, we used a robust spline normalization (RSN), which combines the features of quantile and loess normalization and is designed to normalize the variance-stabilized data. Finally, we identified the potential batch effects and corrected for the RIN, the input cRNA concentration and the barcode of each chip. All microarray data will be available once the article has been published at https://www.ebi.ac.uk/arrayexpress/.

##### Differentially Expressed Genes (DEGs)

To identify differentially expressed genes (DEGs) we used the Linear Models for Microarray Data (Limma) R Package^17^. It estimates the fold changes and standard errors by fitting a linear model for each gene and then, applies empirical Bayes smoothing to the standard errors. We have performed both within-subject and between-subject comparisons. To perform the within-subject comparison, it was estimated for each gene the spatial correlation between the samples from W0 and W1 using the residual maximum likelihood (REML). Then, the between-subject comparisons were performed among the four groups using generalized least squares. We considered as significant those genes with a False Discovery Rate (FDR) lower than 0.05.

DEGs were validated by next generation sequencing using TruSeq® Targeted RNA Expression protocol. A total of 100 ng of RNA were converted to cDNA using ProtoScript II Reverse Transcriptase (New England Biolabs) and libraries were prepared using TruSeq Targeted RNA Expression kit and TruSeq® Targeted RNA custom oligonucleotide pool (Illumina) (Supplementary Table S1). For 21 of the 25 L-H subjects that were included in the microarray analyses after QC, and 7 of the 11 H-L subjects, libraries were pooled and sequenced on the NextSeq 500 instrument (Illumina) using MidOutput v2 kit (150 cycles). The sequencing runs consisted of 100 single-end sequencing cycles. The average % Q30 scores for individual sequencing runs ranged from 89.5–92.0%, with % PF (passing filter) ranging from 82.9–88.92 %. We used the BaseSpace® TruSeq® Targeted RNA v1.0.1 app (Illumina) to analyze the raw data, including alignment to the provided manifest file using a banded Smith Waterman algorithm. This Targeted RNA app includes the following module versions: Isis (Analysis Software): 2.5.57.4.TREx, SAMtools: 0.1.19-isis-1.0.3, Scipy: 0.14.0, Pandas: 0.14.1. The Targeted RNA app generated a target hits file, which displays total reads per amplicon per sample, and this file was imported to R and analyzed using edgeR package^18^.

##### Enrichment analysis

For each probe, we selected only those with FDR < 0.05 from the microarray DEG analyses (comparing the w0 and w1) and calculated a metric based on the -log (raw p-value) and logFC (fold change) in each group (L-H, H-L, L-L and H-H). We uploaded the Entrez gene ID and metric table in WebGestalt (WEB-based Gene SeT AnaLysis Toolket) 2017^19^, selecting as enrich method the Gene Set Enrichment Analysis (GSEA), 1000 permutations, setting the minimum and maximum number of genes in the category as 5 and 2000, respectively, and as collapse method, the mean between duplicate genes. We performed gene ontology (GO) analyses, KEGG (Kyoto Encyclopedia of Genes and Genomes), Panther and Reactome pathway analyses, and FDR <0.05 was considered significant.

##### Co-expression network analysis

Data-driven clustering was performed using weighted gene co-expression network analysis (WGCNA) in each group results comparing *w*0 and *w*1. For this analysis, we used the WGCNA package in R^20^.

### Cross-lagged panel model analysis

Significant DEGs that were exclusively found in the L–H and H–L groups were selected to test bidirectional effects of gene expression and CBCL scores through Cross-Lagged Panel Models (CLPM) with baseline and 3-year follow-up assessments. We used the *lavaan package*^21^ in R and FDR < 0.05 was considered significant.

## Results

From 206 initial samples (103 paired samples), 10 samples from different individuals were excluded during quality control, remaining 196 biological samples. In addition, 10 samples (of these 196) were removed because they had only microarray data from one of the waves (*w*0 or *w*1), which would not allow paired analyses. Thus, 186 samples from 93 participants were included in the final analyses.

There were no statistically significant differences in sex or age (*w*0 or *w*1) among the groups. As expected, the CBCL score at *w*0, *w*1, and ΔCBCL score were significantly different among the groups (Table 1). No statistically significant correlation between CBCL score and age was observed for both waves (*w*0: *r* = −0.013, *p* = 0.890; *w*1: *r* = 0.154; *p* = 0.113) or between ΔCBCL (CBCL w1—CBCL w0) and Δ age (age *w*1—age *w*0) (*r* = −0.154, *p* = 0.113). Moreover, CBCL scores did not increase overtime in the whole BHRCS (*rp* = 0.071; *p*-value = 0.074; Supplementary Fig. S1).

**Table 1:** Demographics of the study population.

Variable	L–H (*n* = 25)	H–L (*n* = 11)	H–H (*n* = 30)	L–L (*n* = 27)	Statistics
Sex (%)	M: 15 F: 10	M: 5 F: 6	M: 19 F: 11	M: 15 F: 12	*χ*^2^ = 1.169; *p* = 0.761
Age in years at *w*0, average (SD)	10.30 (1.67)	10.05 (1.22)	10.02 (1.78)	9.73 (1.54)	*F* = 0.521; *p* = 0.669
Age in years at *w*1, average (SD)	14.12 (1.57)	14.11 (1.21)	13.83 (1.82)	13.46 (1.51)	*F* = 0.856; *p* = 0.467
Total CBCL score at *w*0, average (SD)	18.72(7.69)	48.18 (15.80)	55.83 (20.11)	14.70 (8.10)	*F* = 55.25; *p* = 2.92 × 10^−20^
Total CBCL score at *w*1, average (SD)	47.24 (11.10)	15.54 (9.23)	56.93 (21.18)	15.15 (8.18)	*F* = 51.92; *p* = 1.71 × 10^−19^
ΔCBCL score(SD)	28.52 (9.63)	−32.64 (12.97)	1.10 (6.90)	0.44 (7.12)	*F* = 136.39; *p* = 3.57 × 10^−33^

L–H: low Child Behavior Checklist (CBCL) scores at wave 0 (w0) and high at wave 1 (w1); H–L: high CBCL scores at *w*0 and low at *w*1; H–H: high CBCL scores at w0 and w1; L–L: low CBCL scores at *w*0 and *w*1.

*M* male, *F* female, *SD* standard deviation.

### Differentially expressed genes (DEGs)

**Fig. 1:**
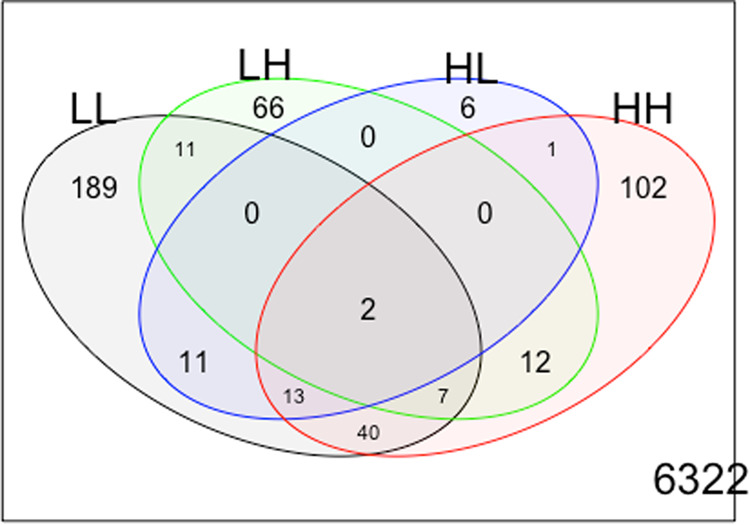
Number of transcripts differentially expressed comparing wave 0 (w0) and wave 1 (w1) for each group. L-L: low CBCL scores at w0 and w1, L-H: low CBCL scores at w0 and high at w1, H-L: high CBCL scores at w0 and low at w1, H-H: high CBCL scores at w0 and w1.

Considering FDR < 0.05, 98 transcripts were differentially expressed in the L–H group (Supplementary Table S2), 33 in the H–L group (Supplementary Table S3), 177 in the H–H group (Supplementary Table S4) and 273 in the L–L group (Supplementary Table S5). Genes that were differentially expressed comparing w0 and w1 in the L–L group are probably related to normal development; and those DEGs in the H–H group might be related to the maintenance of high CBCL scores. Thus, concentrating on genes potentially associated with emergence or remission of psychiatric symptoms, 66 transcripts (and 65 genes, considering that *PEX16* gene had two differentially expressed transcripts) were exclusive of L–H group and six of H–L group (Fig. 1). Of these 71 DEGs, 12 (all from L–H comparison) were correlated with age (Supplementary Table S6), remaining 59 genes that seem to be related to the emergence and remission of general psychiatric symptoms.

We also performed cross-sectional analyses comparing individuals with high psychiatric symptoms (CBCL > 30) with those with low psychiatric symptoms (CBCL ≤ 30) at each wave. However, no significant association was found after multiple comparisons correction (Supplementary Tables S7, S8).

For 51 of the 65 DEGs exclusive of L–H and 4 of the 6 DEGs exclusive of H–L, we validated our results using targeted RNA sequencing data in 21 L–H (out of 25) and 7 H–L (out of 11) subjects. A total of 19.6% (10/51) and 25% (1/4) were significant in both techniques, all of them with similar logFC. However, three genes (*TOP2B, BCOR*, and *GCHFR*) have their expression levels correlated with age. The remaining validated genes are presented in Table 2. Of note, 70.6% (36/51) and all (4/4) were in the same direction in both the microarray and RNA sequencing analyses.

**Table 2:** Differentially expressed genes validated using RNA sequencing.

Gene	logFC	*p*-value
L–H group		
*SMAD4*	−0.372	0.008
*CCM2*	0.209	0.026
*ACSL3*	−0.244	0.045
*CRLF3*	−0.283	0.047
*COX5B*	0.332	0.009
*TSC22D4*	0.246	0.049
*SEC62*	−0.253	0.025
H–L group		
*NDUFA2*	−0.431	0.015

L–H: low Child Behavior Checklist (CBCL) scores at wave 0 (w0) and high at wave 1 (w1); H–L: high CBCL scores at w0 and low at w1; *SMAD4* SMAD Family Member 4, *CCM2* cerebral cavernous malformations 2, *ACSL3* acyl-CoA synthetase long chain family member 3, *CRLF3* cytokine receptor like factor 3, *COX5B* cytochrome C oxidase subunit 5B, *TSC22D4* TSC22 domain family member 4, *SEC62* SEC62 homolog, preprotein translocation factor; *NDUFA2:* NADH: ubiquinone oxidoreductase subunit A2.

### Enrichment and co-expression network analyses

Genes differentially expressed between w0 and w1 in each group were selected for enrichment analyses. No significant enriched category was found for the genes differentially expressed in the H–L or H–H groups. For the L–H group, we found that the Metabolic pathways (hsa01100, FDR = 0.028, normalized enrichment score—NES = 1.97, Supplementary Table S9) in KEGG and the Metabolism pathway in Reactome (R-HAS-1430728, FDR = 0.016, NES = 2.1, Supplementary Table S9) were significantly positively related categories; and, for the L–L group, only Mitochondrial inner membrane (GO:0005743, FDR = 0.049, NES = −1.97, Supplementary Table S10) from GO cellular component was a significantly negative related category. Considering weighted gene co-expression network analysis (WGCNA), no significant co-expression module was detected at any group comparison, after adjusting for multiple comparisons.

### Cross-lagged panel model analyses

We performed cross-lagged panel model analyses for all 65 DEGs exclusive of L–H group and 6 of H–L group using the whole sample. CBCL scores were substantially stable over the course of three years in every cross-lagged model tested (*β* range = 0.606, 0.648; *β* mean = 0.621). Autoregressive analyses revealed that gene expression was only stable for the *TBC1D9, PCNXL4, UBASH3B, NCK2, ETS1, ACSL3*, and *RPS12* genes (Table 3).

**Table 3:** Significant cross-lagged panel models results.

Gene	GENE → CBCL				CBCL → GENE				Autoregressive			
	*β*	*p* value	Adj-*p* value	95% CI	*β*	*p* value	Adj-*p* value	95% CI	*β*	*p* value	Adj-*p* value	95% CI
*TBC1D9*	0.085	0.252	0.677	−0.061, 0.231	0.235	0.006	0.108	0.066, 0.403	0.465	2.009971e−08	1.44718e−06	0.318, 0.613
*PCNXL4*	−0.144	0.120	0.677	−0.259, 0.030	0.182	0.055	0.424	−0.004, 0.367	0.259	0.004	0.041	0.085, 0.433
*RPRD2*	−0.019	0.810	0.972	−0.170, 0.133	0.322	5.664847e−04	0.0407	0.149, 0.495	0.146	0.113	0.313	−0.035, 0.327
*UBASH3B*	0.016	0.826	0.972	−0.129, 0.161	0.098	0.291	0.675	−0.084, 0.281	0.338	1.200074e−04	0.003	0.176, 0.500
*NCK2*	−0.023	0.767	0.972	−0.174, 0.128	0.023	0.816	0.877	−0.172, 0.218	0.271	0.004	0.041	0.089, 0.454
*ETS1*	−0.089	0.238	0.677	−0.237, 0.059	0.065	0.481	0.805	−0.116, 0.247	0.358	6.087803e−05	0.002	0.195, 0.521
*ACSL3*	0.122	0.108	0.677	−0.027, 0.271	−0.086	0.367	0.684	−0.273, 0.101	0.290	1.714824e−03	0.025	0.116, 0.463
*RPS12*	−0.103	0.175	0.677	−0.252, 0.046	−0.045	0.638	0.835	−0.233, 0.143	0.289	1.22921e−03	0.022	0.114, 0.464

*CBCL* child behavior checklist, *TBC1D9* TBC1 domain family member 9, *PCNXL4* Pecanex 4, *RPRD2* regulation of nuclear pre-MRNA domain containing 2, *UBASH3B* ubiquitin associated and SH3 domain containing B, *NCK2* NCK adapter protein 2, *ETS1*: ETS proto-oncogene 1, transcription factor, *ACSL3* acyl-CoA synthetase long chain family member 3, *RPS12* ribosomal protein S12.

Longitudinal analyses showed that CBCL scores at *w*0 predicted the expression of the *RPRD2* gene at w1 (Table 3); however, no gene at w0 was associated with CBCL scores at *w*1 after adjustment for multiple comparisons. Results from all cross-lagged analyses are described in Supplementary Table S11.

## Discussion

This study found differentially expressed genes in blood, related to different psychopathological trajectories during childhood and adolescence, focusing on the emergence and remission of psychiatric symptoms in a 3-year follow-up. Comparing w0 and w1, we found 98 transcripts differentially expressed in the L–H group, 33 in the H–L group, 177 in the H–H group and 273 in the L–L group. DEGs found in the last two groups might be related to age, whereas those found in the first two are suggested here as associated to changes in psychopathology. Although others analyzed shared blood and brain transcriptomes in different major mental disorders or the conversion to specific mental disorders, no other study has investigated gene expression and changes in psychopathology during childhood and adolescence.

### DEGs comparing w0 and w1 in the H–H and L–L groups

A total of 55 DEGs (of 177) in the H–H group and 74 (of 273) in the L–L group (30 were in common for both groups) were associated with age in the discovery stage of a previous study^22^. *RSRC2* (*Arginine And Serine Rich Coiled-Coil 2*) and *NUP160* (*Nucleoporin 16*0), both differentially expressed in the H–H and L–L groups, were previously correlated with age in peripheral blood leukocytes^23^. Other genes included *S100A4* (*S100 Calcium Binding Protein A4*), which was elevated in w1 in L–L, was previously reported as overexpressed with age^24^. Also, at least four genes in the L–L group (*NDUFS5 -NADH:Ubiquinone Oxidoreductase Subunit S5, PDCD6IP*—*Programmed Cell Death 6 Interacting Protein*, *SEC61G*—*Sec61 Translocon Gamma Subunit* and *STX10*—*Syntaxin 10*) and one in the H–H group (*TMSL3*, or *TMSB4XP8*—*TMSB4X Pseudogene 8*) contain or are near CpG sites considered DNA methylation age predictors^25^.

Of note, Gandal et al.^11^ analyzed microarray data from postmortem brain and identified one module annotated as mitochondrial inner membrane and cellular respiration as downregulated across ASD, schizophrenia and bipolar disorder^11^. This same pathway was downregulated in our L–L group, suggesting that this pattern might be associated with normal development as well.

We consider that differentially regulated genes in the H–H or L–L groups are probably related to normal trajectory of development, puberty, though some previous studies did not associate these genes with it^26,27^, or to the maintenance of the symptoms of chronic mental disorders or at least not related to a change of status concerning mental disorders. Thus, we focused on genes that were exclusively differentially expressed in the L–H and H–L groups.

### DEGs comparing w0 and w1 in the L–H group

A total of 66 transcripts (and 65 genes) seems to be related to the emergence of psychopathology; however, none was among the most significant differentially expressed genes in previous studies that investigated shared gene expression patterns in cross-disorder analyses^10,11,28,29^. Moreover, only the expression of *TBC1D9, PCNXL4, UBASH3B, NCK2, ETS1*, and *ACSL3* seems to be stable over the course of three years. Interestingly, cross-lagged analyses revealed that the expression of *RPRD2* (Regulation Of Nuclear Pre-MRNA Domain Containing 2) at w1 can be influenced by CBCL at w0, though very few studies have investigated this gene and none in mental disorders. Among the 65 DEGs, 12 were correlated with age (Supplementary Table S6), and might reflect age-related genes, even though we excluded those DEGs in L–L group. Notably, 10 of 51 DEGs were also validated by another technique, though three were related to age and were not presented in Table 2.

Differentially expressed genes in the L–H group seem to be enriched for metabolic pathways. Of note, *COX5B* (Cytochrome C Oxidase Subunit 5B, adjusted *p* = 0.034, logFC = 0.271), which was also validated by RNA sequencing, encodes one of the nuclear-coded polypeptide chains of cytochrome c oxidase, the terminal oxidase in mitochondrial electron transport. Its protein levels seem to be increased in resilience to stress in a rat model of depression^30^. Interestingly, it is differentially expressed in postmortem brain of subjects with ASD or schizophrenia in the study of Gandal et al.^11^, which performed a meta-analysis of transcriptomic studies^11^. In the same metabolic pathway, *SIN3A* (SIN3 Transcription Regulator Family Member A, adjusted *p* = 0.048, logFC = −0.184), although not significantly validated (*p* = 0.062; logFC = −0.253), seems to have a trend to decrease its expression levels with the increase of psychiatric symptoms. This gene encodes a transcriptional repressor that seems to play a role in cell cycle and proliferation. Witteveen et al.^31^ found that its haploinsufficiency is associated with mild syndromic intellectual disability and that SIN3A is essential for cortical brain development^31^. Additionally, SIN3A seems to act as a corepressor for RE-1 silencing transcription factor (REST), which is also known as neuron-restrictive silencer factor (NRSF). Therefore, *SIN3A* is a potential gene to play a role in the emergence of psychiatric symptoms, considering that REST/NRSF is known to regulates neurogenesis and neural differentiation^32^.

Although *S100A6* (S100 Calcium Binding Protein A6, adjusted *p* = 0.042, logFC = 0.357) was not analyzed in the RNA sequencing, it seems to be an interesting gene. It encodes a member of the S100 family of proteins containing 2 EF-hand calcium-binding motifs and seems to be involved in cellular calcium signaling. Its expression levels were increased in high hallucinations states^33^; however, another study identified a lower expression in peripheral blood lymphocytes from schizophrenia patients compared to controls^34^.

*TSC22D4* (TSC22 Domain Family Member 4, adjusted *p* = 0.038, logFC = 0.276) is a member of the TSC22 domain family of leucine zipper transcriptional regulators. Although few studies investigated this gene, Pfaffenseller et al., investigating differential expression of transcriptional regulatory units in the prefrontal cortex of patients with bipolar disorder (BD), identified that *TSC22D4* regulatory unit was increased in BD^35^. Moreover, a SNP near this gene, rs2406253, was associated to self-reported educational attainment in multiple genome-wide association studies^36,37^.

*CCM2* gene (Cerebral Cavernous Malformations 2, adjusted *p* = 0.022, logFC = 0.331) encodes a scaffold protein that functions in the stress-activated p38 Mitogen-activated protein kinase (MAPK) signaling cascade. Mutations in this gene have been associated to cerebral cavernous malformations, which are brain vascular lesions that may lead to neurologic problems^38^. Although it has been consistently related to this disorder, studies investigating the role of this gene in other disorders are scarce. The peripheral blood expression of *CCM2* seems to be related to chronic academic stress in healthy medical students^39^.

*SMAD4* gene (SMAD Family Member 4, adjusted *p* = 0.022, logFC = −0.182) encodes a protein involved in signal transduction of the transforming growth factor-beta superfamily (TGFB) and bone morphogenic proteins (BMP). Mutations in this gene are related to Myhre syndrome, a connective tissue disorder with multisystem involvement with or without intellectual disability. Cases with this disorder and *SMAD4* mutation and ASD have been described^40^ and a SNP within this gene was associated with psychosis in Korean families^41^. This gene seems to play a role in neuronal differentiation^42^ and cerebellar development^43^.

*CRLF3* (Cytokine Receptor Like Factor 3, adjusted *p* = 0.034, logFC = −0.187) encodes a largely uncharacterized orphan cytokine receptor that is expressed in various human tissues, including brain. Its function is not well known; however, it has been associated with cell cycle regulation, neuronal morphology, and amyotrophic lateral sclerosis^44^.

*SEC62* (SEC62 Homolog, Preprotein Translocation Factor, adjusted *p* = 0.047, logFC = −0.192) was suggested to play a role in protein translocation, calcium homeostasis and the recovery from endoplasmic reticulum stress^45^. Although no study investigated this gene in mental disorders, *SEC62* was among the differentially expressed genes in ASD brain samples, showing decreased transcript levels^11^.

### DEGs comparing w0 and w1 in the H–L group

Six genes were associated with the remission of psychiatric symptoms, with *NDUFA2* being the most significant (adjusted *p*-value = 0.014, logFC = −0.466) and the only one validated with another technique (Table 2). *NDUFA2* (NADH: Ubiquinone Oxidoreductase Subunit A2) encodes a subunit of the hydrophobic protein fraction of the NADH: ubiquinone oxidoreductase (complex 1), the first enzyme complex in the electron transport chain located in the inner mitochondrial membrane. This gene seems to be upregulated in the prefrontal cortex of an adolescent social isolation, a typical paradigm for schizophrenia, rat model^46^, in line with our results that showed a downregulation in the remission of psychiatric symptoms. Moreover, *NDUFA2* was among the differentially expressed genes in postmortem schizophrenia brain samples^11^.

### Strengths and limitations

Our study presents some strengths and limitations that deserve attention. First, our study design was longitudinal, and it combined the collection of clinical assessment and RNA samples at baseline and at a 3 years follow-up; this can help to elucidate the temporality of the relationship between the development of psychopathology and gene expression changes. Second, psychopathology exists on a continuum in the general population, and population-based studies have demonstrated that symptoms, when considered dimensionally, vary with neurobiological features^47,48^, providing further support for the examination of dimensional psychopathology. Third, we studied youths, a much less studied population than adults, even though the psychopathology could be severe, persistent, and with a significant impact over the functional trajectory across the life. Moreover, psychiatric symptomatology during childhood and adolescence predicts persistent mental illness later in life^49^.

The results of this study should be interpreted at light of some limitations. First the relatively small sample size could be an issue, thus, further replication studies are needed. Moreover, even not validated by RNA sequencing, some of the DEGs found in the microarray might be interesting to be replicated in other studies with increased sample sizes, such as *PGAM1* and *BCL2*, which have been associated to mental disorders and were in the same direction of association in the RNA sequencing and microarray. Second, gene expression is tissue-specific, and our findings may not mirror gene expression changes in brain, though some of our results were also found in a meta-analysis of human postmortem brain transcriptomic studies^11^. We would also like to highlight that we were focused in searching potential peripheral markers. Furthermore, it is unknown whether the gene expression differences observed in this study of blood samples will remain stable over time or will change at the onset of a full-blown mental disorder, since RNA levels are dynamic throughout development. However, the low stability and high comorbidity patterns of categorical psychopathology in this age range, as assessed by our current classification (e.g., DSM), support our approach to psychopathology. Finally, it is not possible to know if other factors that may change gene expression influenced our results, such as body mass index, or physical exercise.

## Conclusions

In conclusion, we identified 72 transcripts from 71 genes related to the emergence and remission of general psychiatric symptoms during adolescence, though 12 might be age-related. A set of genes seems to be related to metabolic processes, neurodevelopment and mental disorders. One of them (*RPRD2*) might have its expression at w1 influenced by the CBCL scores at w0. Three (*COX5B, SEC62*, and *NDUFA2*) were validated with another technique and were also differentially regulated in postmortem brain of subjects with mental disorders. Our findings support the further exploration of changes in transcription of these set of genes as peripheral markers of emergence or remission of mental disorders and their dimensions.

## Supplementary information

Suplementary Figure S1

Suplementary Tables

## References

[1] Copeland WE (2013). Diagnostic transitions from childhood to adolescence to early adulthood. J. Child Psychol. Psychiatry Allied Discip..

[2] Martel MM (2017). A general psychopathology factor (P factor) in children: structural model analysis and external validation through familial risk and child global executive function. J. Abnorm. Psychol..

[3] Sullivan PF, Daly MJ, O’Donovan M (2012). Genetic architectures of psychiatric disorders: the emerging picture and its implications. Nat. Rev. Genet..

[4] Franke B (2012). The genetics of attention deficit/hyperactivity disorder in adults, a review. Mol. Psychiatry.

[5] Smoller JW, Finn CT (2003). Family, twin, and adoption studies of bipolar disorder. Am. J. Med. Genet. C..

[6] Lichtenstein P, Carlstrom E, Rastam M, Gillberg C, Anckarsater H (2010). The genetics of autism spectrum disorders and related neuropsychiatric disorders in childhood. Am. J. Psychiatry.

[7] Cross-Disorder Group of the Psychiatric Genomics Consortium. (2013). Identification of risk loci with shared effects on five major psychiatric disorders: a genome-wide analysis. Lancet..

[8] Cross-Disorder Group of the Psychiatric Genomics Consortium. (2019). Genomic relationships, novel loci, and pleiotropic mechanisms across eight psychiatric disorders. Cell.

[9] Rice F (2019). Characterizing developmental trajectories and the role of neuropsychiatric genetic risk variants in early-onset depression. JAMA Psychiatry.

[10] de Jong S (2016). Immune signatures and disorder-specific patterns in a cross-disorder gene expression analysis. Br. J. Psychiatry.

[11] Gandal MJ (2018). Shared molecular neuropathology across major psychiatric disorders parallels polygenic overlap. Science.

[12] Salum GA (2015). High risk cohort study for psychiatric disorders in childhood: rationale, design, methods and preliminary results. Int. J. Methods Psychiatr. Res..

[13] Achembach TM, Rescorla LA (2001). Manual for the ASEBA School-Age Forms & Profiles.

[14] Spindola LM (2019). Detecting multiple differentially methylated CpG sites and regions related to dimensional psychopathology in youths. Clin. Epigenet..

[15] Du P, Kibbe WA, Lin SM (2008). lumi: a pipeline for processing Illumina microarray. Bioinformatics.

[16] Allen JD, Chen M, Xie Y (2009). Model-based background correction (MBCB): R methods and GUI for illumina bead-array data. J. Cancer Sci. Ther..

[17] Ritchie ME (2015). limma powers differential expression analyses for RNA-sequencing and microarray studies. Nucleic Acids Res..

[18] Robinson MD, McCarthy DJ, Smyth GK (2010). edgeR: a Bioconductor package for differential expression analysis of digital gene expression data. Bioinformatics.

[19] Wang J, Vasaikar S, Shi Z, Greer M, Zhang B (2017). WebGestalt 2017: a more comprehensive, powerful, flexible and interactive gene set enrichment analysis toolkit. Nucleic Acids Res..

[20] Langfelder P, Horvath S (2008). WGCNA: an R package for weighted correlation network analysis. BMC Bioinform..

[21] Rosseel, Y. lavaan: an R package for structural equation modeling. *J. Stat. Softw.* (2012). 10.18637/jss.v048.i02.

[22] Peters MJ (2015). The transcriptional landscape of age in human peripheral blood. Nat. Commun..

[23] Irizar H (2015). Age gene expression and coexpression progressive signatures in peripheral blood leukocytes. Exp. Gerontol..

[24] de Magalhaes JP, Curado J, Church GM (2009). Meta-analysis of age-related gene expression profiles identifies common signatures of aging. Bioinformatics.

[25] Horvath S (2013). DNA methylation age of human tissues and cell types. Genome Biol..

[26] Hou H (2017). Gene expression profiling of puberty-associated genes reveals abundant tissue and sex-specific changes across postnatal development. Hum. Mol. Genet..

[27] Thompson EE (2018). Global DNA methylation changes spanning puberty are near predicted estrogen-responsive genes and enriched for genes involved in endocrine and immune processes. Clin. Epigenet..

[28] Ellis SE, Panitch R, West AB, Arking DE (2016). Transcriptome analysis of cortical tissue reveals shared sets of downregulated genes in autism and schizophrenia. Transl. Psychiatry.

[29] Zhao Z (2015). Transcriptome sequencing and genome-wide association analyses reveal lysosomal function and actin cytoskeleton remodeling in schizophrenia and bipolar disorder. Mol. Psychiatry.

[30] Henningsen K (2012). Candidate hippocampal biomarkers of susceptibility and resilience to stress in a rat model of depression. Mol. Cell Proteom..

[31] Witteveen JS (2016). Haploinsufficiency of MeCP2-interacting transcriptional co-repressor SIN3A causes mild intellectual disability by affecting the development of cortical integrity. Nat. Genet..

[32] Song Z, Zhao D, Zhao H, Yang L (2015). NRSF: an angel or a devil in neurogenesis and neurological diseases. J. Mol. Neurosci..

[33] Kurian SM (2011). Identification of blood biomarkers for psychosis using convergent functional genomics. Mol. Psychiatry.

[34] Bowden NA (2006). Preliminary investigation of gene expression profiles in peripheral blood lymphocytes in schizophrenia. Schizophr. Res..

[35] Pfaffenseller B (2016). Differential expression of transcriptional regulatory units in the prefrontal cortex of patients with bipolar disorder: potential role of early growth response gene 3. Transl. Psychiatry.

[36] Okbay A (2016). Genome-wide association study identifies 74 loci associated with educational attainment. Nature.

[37] Lee JJ (2018). Gene discovery and polygenic prediction from a genome-wide association study of educational attainment in 1.1 million individuals. Nat. Genet..

[38] Chohan MO (2019). Emerging pharmacologic targets in cerebral cavernous malformation and potential strategies to alter the natural history of a difficult disease: a review. JAMA Neurol..

[39] Honda M (2013). Chronic academic stress increases a group of microRNAs in peripheral blood. PLoS ONE.

[40] Artemios P (2019). Autism spectrum disorder and psychiatric comorbidity in a patient with Myhre syndrome. J. Autism Dev. Disord..

[41] Cho MJ, Lee BD, Kim C (2015). Pilot study for family-based association analysis of schizophrenia in a Korean population: analysis for candidate genes positionally on chromosome 18q21. Asia Pac. Psychiatry.

[42] Kawaguchi-Niida M, Shibata N, Furuta Y (2017). Smad4 is essential for directional progression from committed neural progenitor cells through neuronal differentiation in the postnatal mouse brain. Mol. Cell Neurosci..

[43] Fernandes M, Antoine M, Hebert JM (2012). SMAD4 is essential for generating subtypes of neurons during cerebellar development. Dev. Biol..

[44] Hahn N (2019). The orphan cytokine receptor CRLF3 emerged with the origin of the nervous system and Is a neuroprotective erythropoietin receptor in locusts. Front Mol. Neurosci..

[45] Linxweiler M, Schick B, Zimmermann R (2017). Let’s talk about Secs: Sec61, Sec62 and Sec63 in signal transduction, oncology and personalized medicine. Signal Transduct. Target Ther..

[46] Sun L, Min L, Li M, Shao F, Wang W (2018). Transcriptomic analysis reveals oxidative phosphorylation activation in an adolescent social isolation rat model. Brain Res. Bull..

[47] Blanken LM (2015). Cortical morphology in 6- to 10-year old children with autistic traits: a population-based neuroimaging study. Am. J. Psychiatry.

[48] Mous SE (2014). Cortical thickness and inattention/hyperactivity symptoms in young children: a population-based study. Psychol. Med..

[49] Hofstra MB, van der Ende J, Verhulst FC (2002). Child and adolescent problems predict DSM-IV disorders in adulthood: a 14-year follow-up of a Dutch epidemiological sample. J. Am. Acad. Child Adolesc. Psychiatry.

