# Supplementary material for: Gene expression changes associated with trajectories of psychopathology in a longitudinal cohort of children and adolescents
Source: Transl Psychiatry. 2020 Mar 17;10:99. doi: 10.1038/s41398-020-0772-3 (PMC7078305; doi:10.1038/s41398-020-0772-3)

**Supplementary Figure S1: Scatter plot of CBCL total score and chronological age in all subjects from the Brazilian High Risk Cohort Study (BHRCS) who have blood collected in both wave 0 and wave 1 (n=319 individuals; 638 biological samples).**


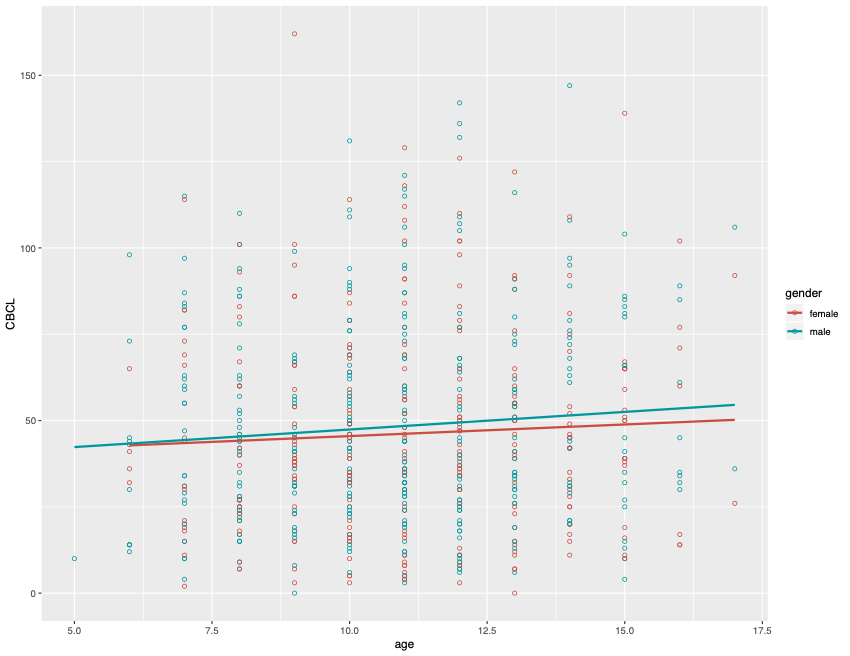

Supplement: Supplementary file 1 — Suplementary Figure S1 [file 41398_2020_772_MOESM1_ESM.docx]
